# Poly(I:C) transfection induces a pro-inflammatory cascade in murine mammary carcinoma and fibrosarcoma cells

**DOI:** 10.1080/15476286.2022.2084861

**Published:** 2022-06-23

**Authors:** A Sales Conniff, G Encalada, S Patel, M Bhandary, F Al-Takrouri, L Heller

**Affiliations:** Department of Medical Engineering, University of South Florida, Tampa, FL, USA

**Keywords:** Cancer, double-stranded RNA (dsRNA), polyinosinic-polycytidylic acid, poly(I:C), pattern recognition receptors PRRs, RNA sensors, electroporation

## Abstract

Germline-encoded pattern recognition receptors [PRRs] in mammalian cells function in the detection of molecular patterns associated with pathogen invasion or cellular damage. A PRR subset is activated by the atypical presence and location of double-stranded RNA [dsRNA] or its synthetic analogue polyinosinic-polycytidylic acid [poly(I:C)], triggering pro-inflammatory signalling and death in many cell types. Poly(I:C) has been tested as a sole or combination cancer therapy in preclinical studies and clinical trials. The purpose of this study was to evaluate the effects of poly(I:C) transfection via electroporation on cell lines from a cancer of epithelial origin, 4T1 mammary carcinoma, and a cancer of mesenchymal origin, WEHI 164 fibrosarcoma. The effects of the poly(I:C) delivery on cell metabolism implicate the induction of cell death. A pro-inflammatory response was demonstrated by mRNA upregulation and the secretion of Type I interferon and several cytokines and chemokines. The mRNAs of dsRNA sensor DExD/H-box helicase 58/retinoic acid-inducible gene I protein [Ddx58/RIG-I] and sensor/co-sensor DEAH-box helicase 9 [Dhx9] were not regulated, but the mRNAs of RNA sensors toll-like receptor 3 [TLR3], interferon-induced with helicase C domain 1/melanoma differentiation-associated protein 5 [Ifih1/MDA5] and Z-DNA binding protein 1 [Zbp1] and co-sensors DEAD (Asp-Glu-Ala-Asp) box polypeptide 60 [Ddx60] and interferon-inducible protein 204 [Ifi204] were upregulated in both cell lines. The mRNAs encoding signalling pathways components were present or upregulated in both cell types. These data demonstrate that RNA sensing effects can be amplified by electroporation delivery, potentially expanding the practicality of this immunotherapeutic approach.

## Introduction

Pattern recognition receptors (PRRs) specifically recognize molecular patterns associated with pathogen invasion or cell damage. Based on their protein domain homology in vertebrates, PRRs are classified into toll-like receptors [TLRs], nucleotide oligomerization domain (NOD)-like receptors [NLRs], retinoic acid-inducible gene-I (RIG-I)-like receptors [RLRs], C-type lectin receptors [CLRs], and absent in melanoma-2 (AIM2)-like receptors [ALRs] [1], although several PPRs are unassigned. Double-stranded RNA (dsRNA) sensors such as TLR3 [2], DExD/H-box helicase 58/retinoic acid-inducible gene I protein [Ddx58/RIG-I] [3,4], interferon-induced with helicase C domain 1/melanoma differentiation-associated protein 5 [Ifih1/MDA5] [3] and Z-DNA binding protein 1 [Zbp1] [5] discriminate between mammalian and foreign RNA in the endosomes and cytosol [6]. However, the complete understanding of RNA sensing is evolving. The putative DNA sensors DEAD (Asp-Glu-Ala-Asp) box polypeptide 60 [Ddx60] and DEAH-box helicase 9 [Dhx9] [7,8] and interferon-inducible protein 16 [Ifi16], the human ortholog of mouse Ifi204 [9], also sense dsRNA and act as co-sensors [10]. These sensors signal via overlapping pathways to induce the secretion of Type I interferons, pro-inflammatory cytokines and chemokines, and cell death [6,10].

Following traditional cytotoxic therapies such as chemotherapy and radiotherapy, cancer cells can exhibit remarkable resistance to treatment due to multiple factors, including an immune-suppressive tumour microenvironment. Therefore, immunotherapy has emerged as standard combination cancer therapy. The innate immunity induced by RNA sensing may potentiate adaptive anticancer immunity [11] so RNA sensors are emerging therapeutic targets [12–14]. Sole and combined cancer therapies including polyinosinic-polycytidylic acid [poly (I:C)] and its derivatives are in clinical trials for many cancer types, including breast cancer and soft tissue sarcoma [13–15].

Several molecular delivery systems have been developed to improve the therapeutic index and cellular uptake to lower the dosage of the molecule [16]. Electroporation is a molecular delivery method based on the application of defined electric pulses to cells or tissues, leading to increased cell membrane permeability and the transport of nucleic acids or drugs into cells [17]. Reversible electroporation is temporary; cells ultimately repair the plasma membrane and re-establish homoeostasis. This delivery method is clinically approved in several European countries for the enhanced intratumour delivery of chemotherapeutic agents [18,19]. Worldwide, gene therapy clinical trials are in progress for cancer therapies and infectious disease vaccines using electroporation delivery [20,21]. We chose this simple method to deliver poly(I:C), a synthetic analogue of dsRNA, into 4T1 mammary carcinoma and WEHI 164 fibrosarcoma cells to assess the amplification of potential adjuvant effects.

## Materials and Methods

### Cell lines

4T1 mouse mammary cancer cells (CRL2539, American Type Culture Collection (ATCC), Manassas, VA, USA) were cultured in ATCC formulated Roswell Park Memorial Institute Medium (RPMI-1640) supplemented with 5% foetal bovine serum (FBS, Gibco, Waltham, MA, USA). WEHI 164 murine fibrosarcoma cells (ATCC CRL-1751) were cultured in RPMI-1640 (Gibco) supplemented with 5% FBS. All cells were cultured in a 5% CO_2_ humidified atmosphere at 37°C. Cells were regularly tested for the presence of *Mycoplasma* spp. with the Myco-Sniff PCR Detection Kit (MP Biochemicals, Irvine, CA, USA) and found negative.

### Transfection

Poly (I:C) (Tocris Bioscience, Bristol, UK) was suspended to 2 mg/ml in molecular grade water. Cells were suspended to a concentration of 2.5^10^7^/ml in culture medium with a final concentration of 0.4 mg/ml poly(I:C). Six 100 μs pulses with a voltage to distance ratio of 1300 V/cm and a frequency of 4 Hz [22] were applied in cuvettes using an ECM 830 square wave electroporation system (BTX Harvard Apparatus, Holliston, MA, USA).

### *Cell viability measured by metabolic assay* in vitro

Twenty-four hours after transfection, the medium was replaced with 100 µl medium containing PrestoBlue (Invitrogen, Thermo Fisher Scientific), incubated for 1 hour, and viability quantified as determined by fluorescence intensity with a CLARIOstar microplate reader (BMG Labtech, Cary, NC, USA). The survival of cells in the experimental groups was normalized to the control group.

### RT-qPCR (Reverse transcription-quantitative polymerase chain reaction)

Total RNA was extracted from cells 4 hours after transfection using Trizol Reagent (Invitrogen, Thermo Fisher Scientific) then purified on RNeasy columns (Qiagen, Valencia, CA). After quantification, 250 ng of total RNA was reverse transcribed into complementary DNA (SuperScript II Reverse Transcriptase, Invitrogen, Thermo Fisher Scientific) according to the manufacturer’s instructions and diluted 10-fold. Messenger RNA was quantified on an Applied Biosystems QuantStudio 3 real-time thermal cycler (Thermo Fisher Scientific, Waltham, MA, USA) using custom primers (Integrated DNA Technologies, Coralville, IA) in SYBR Green Master Mix (Applied Biosystems, Thermo Fisher Scientific). Primer sequences are listed in Supplementary Table S1. Spleen RNA acted as a positive control. Relative quantification was performed by comparison to the reference genes β-actin and glyceraldehyde 3-phosphate dehydrogenase using the ΔΔCt method [23].

### Protein quantification by ELISA

The secretion of the pro-inflammatory proteins interferon beta (IFNβ, VeriKine-HS Mouse Interferon Beta Serum ELISA Kit, PBL Assay Science, Piscataway, NJ, USA) and interleukin-6 (IL-6, Quantikine IL-6 ELISA, R&D Systems, Minneapolis, MN, USA), into the medium was measured 4 hours after poly(I:C) transfection according to the manufacturer’s instructions.

### Protein quantification by bead array

The total protein concentration in the medium was normalized and 25 µg of each sample was analysed using a premixed multiplex for CCL2, CCL4, CCL3 and CXCL10 (Mouse Cytokine/Chemokine Magnetic Luminex Assay, Millipore, Burlington, MA, USA) on a MAGPIX System (Luminex, Austin, TX, USA) per manufacturer’s instructions.

### Protein quantification by western blot

Four hours after poly(I:C) electroporation, cells were lysed in RIPA buffer per the manufacturer’s instructions (Santa Cruz Biotechnology Inc., Dallas, TX, USA). Protein concentrations of the lysates were measured using a Pierce BCA Protein Assay Kit (Thermo Fisher Scientific) then adjusted with RIPA buffer. Equal amounts of protein were loaded into the wells of a 10% acrylamide SDS-PAGE gel and the separated proteins transferred to nitrocellulose membranes (Bio-Rad Laboratories, Inc, Hercules, CA, USA). The membranes were blocked with 3% (w/v) bovine serum albumin in tris buffered saline and then incubated overnight at 4°C with primary antibodies diluted 1:500 (Phospho-IRF-7 (Ser477) (D7E1W) Rabbit mAb #12390, Cell Signaling Technologies, Danvers, MA, USA), p-IRF3 (PA5-38,285, Invitrogen, Thermo Fisher Scientific). The membranes were washed with phosphate buffered saline Tween (#28360, Thermo Fisher Scientific) and incubated with a horseradish peroxidase-conjugated rabbit anti-mouse IgG (diluted 1:2500; Promega, Madison, WI) for 30 min at room temperature. The membrane was then exposed to HRP chemiluminescent substrate (#1705060, Clarity Western ECL Substrate, Bio-Rad, Hercules, CA, USA) and the protein bands on the gels and membranes were visualized after separation and transfer using the signal accumulation mode in a ChemiDoc MP System (Bio-Rad, Hercules, CA, USA). Image Lab (version 6.1, Bio-Rad) was used to measure the adjusted intensity of the total protein bands after SDS-PAGE and the chemiluminescent signals from the western blots and intensity quantified by Image J software [24].

### Statistical Analysis

GraphPad Prism 9.1.0 (San Diego, CA, USA) was used for statistical analysis and graph preparation. Since the data were normally distributed, significance was determined by a two-tailed t-test or one-way ANOVA test followed by a Tukey-Kramer post-test. A p < 0.05 was considered statistically significant.

## Results

### Poly(I:C) transfection and cell viability

**Figure 1.**
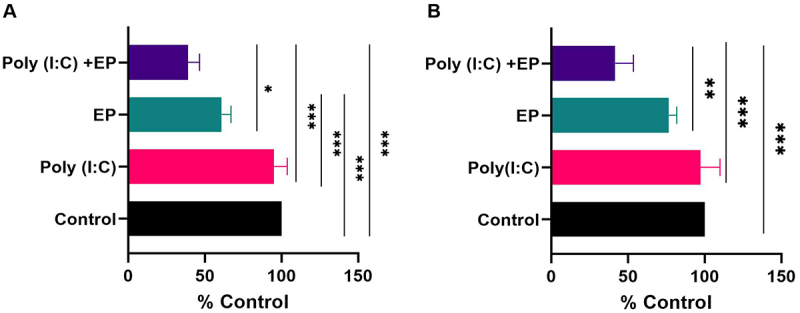
Cell metabolism measured 24 hours after poly(I:C) transfection normalized to the control group in (A) mammary carcinoma cells and (B) fibrosarcoma cells. (***p < 0.001, **p < 0.01, * p < 0.05 compared to control, n = 3). EP, electroporation.

We initially examined the viability of both mammary carcinoma and fibrosarcoma cells 24 hours after the delivery of poly(I:C) into the cells using a metabolic assay (Fig. 1). No statistically significant effect on viability was observed with poly(I:C) exposure. Pulse application alone affected the viability of mammary carcinoma cells (p < 0.001) but did not affect fibrosarcoma cell viability when compared to the control group. Finally, poly(I:C) transfection substantially decreased the cell viability of both cell lines when compared to control cells (p < 0.001).

### Poly(I:C) transfection induces upregulation of pro-inflammatory cytokines and chemokines

**Figure 2.**
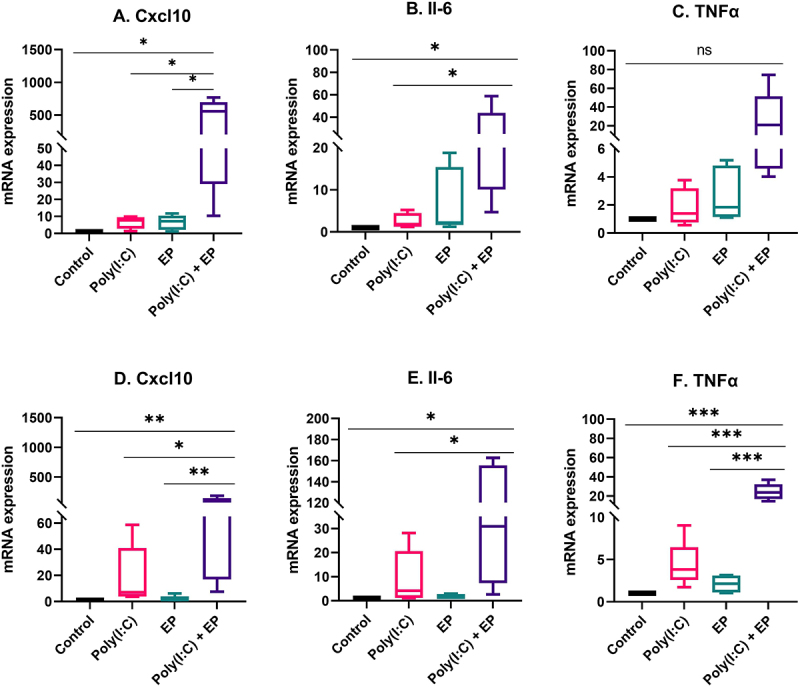
Regulation of pro-inflammatory mRNAs by poly(I:C) transfection. Relative mRNA levels (A) CXCL10, (B) IL-6 and (C) TNFα mRNA in mammary carcinoma cells; (D) CXCL10, (E) IL-6, and (F) TNFα mRNA in fibrosarcoma cells. (***p < 0.001, **p < 0.01, * p < 0.05 compared to control, n = 3–5).

We then compared the cell line responses to transfection of this dsRNA analogue. After 4 hours, we quantified the relative mRNA expression of pro-inflammatory cytokines and chemokines by RT-qPCR (Fig. 2). Neither poly(I:C) exposure nor pulse application alone significantly affected Cxcl10, IL-6, and TNFα mRNAs mRNA levels in either cell line (Fig. 2A-F). However, mammary carcinoma cells responded to poly(I:C) transfection with the upregulation of Cxcl10 and IL-6 but not TNFα mRNA levels (Fig. 2A-C). In fibrosarcoma cells, the levels of each of these mRNAs were significantly upregulated (Fig. 2D-F).

**Figure 3.**
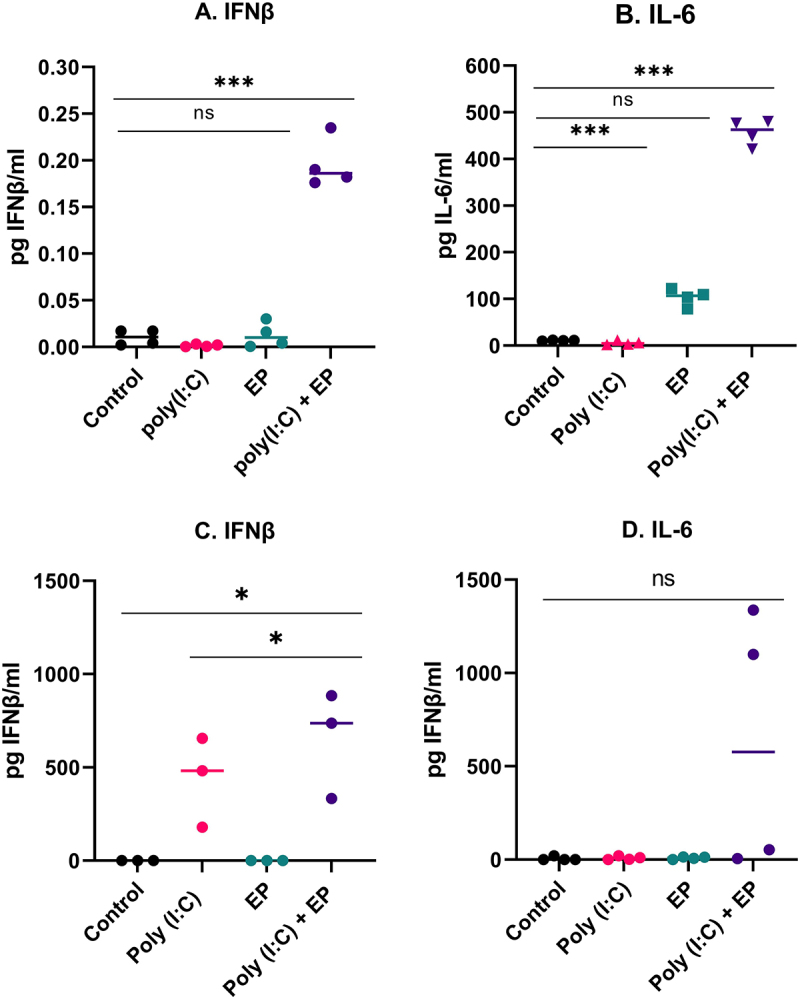
Protein secreted into the medium as determined by ELISA four hours after poly(I:C) transfection (A, B) by mammary carcinoma cells or (C, D) by fibrosarcoma cells (***p < 0.001, * p < 0.05 compared to control, ns = non-significant, n = 3–4).

We next sought to evaluate the profiles of the IFNβ and IL-6 protein secretion by ELISA. After poly(I:C) transfection, the levels of protein expression of IFNβ and IL-6 on mammary carcinoma cells were significantly upregulated (Fig. 3A, B). We observed a high level of IFNβ secretion by the fibrosarcoma cells (Fig. 3C). Secretion of IL-6 tended to be upregulated on the fibrosarcoma cells electroporated with poly(I:C) but was not statistically significant (Fig. 3D).

**Figure 4.**
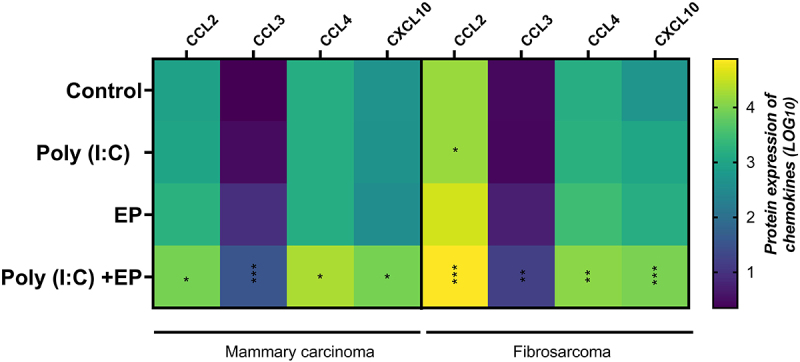
Chemokines secreted into the medium as determined by bead array four hours after poly(I:C) transfection of mammary carcinoma or fibrosarcoma cells. Fold expression is shown in a Log_10_ scale. (***p < 0.001, **p < 0.01, * p < 0.05 compared to control, n = 5). Chemokine (C-C motif) ligand 2 [CCL2]; Chemokine (C-C motif) ligand 3 [CCL3]; Chemokine (C-C motif) ligand 4 [CCL4]; C-X-C motif chemokine ligand 10 [CXCL10].

The expression of many pro-inflammatory proteins is induced by RNA sensing [6,10]. Therefore, we also assayed for the secretion of several pro-inflammatory chemokines (Fig. 4). Fibrosarcoma cells responded to poly(I:C) exposure with significant production of CCL2 (p < 0.05) that was amplified with transfection. In both the mammary carcinoma and fibrosarcoma cells, secretion of CCL2, CCL3, CCL4, and Cxcl10 was upregulated after poly(I:C) transfection.

### RNA sensors mRNAs are expressed and regulated in mammary carcinoma and fibrosarcoma cells

**Figure 5.**
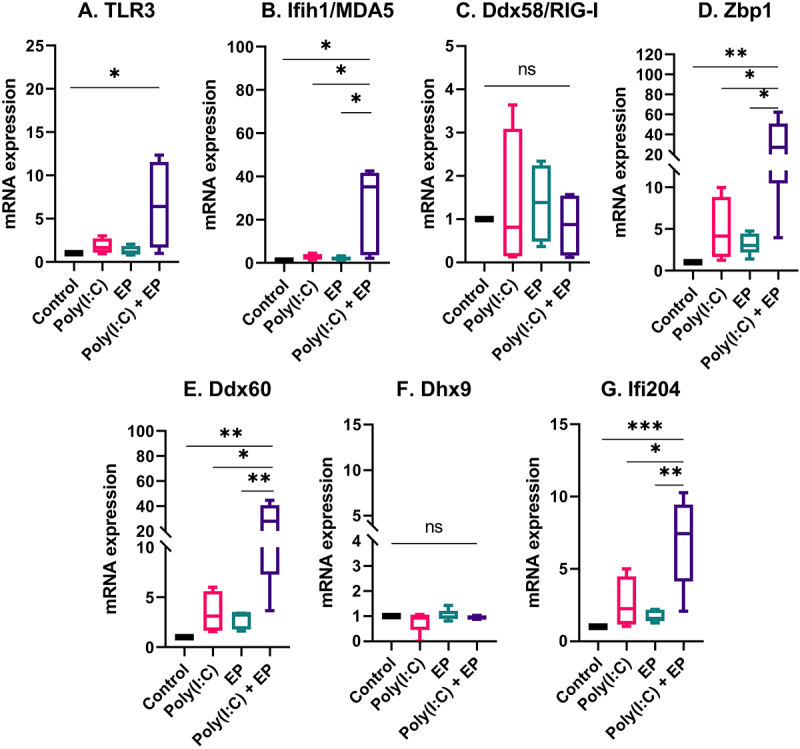
The regulation of several dsRNA sensor and co-sensor mRNAs by poly(I:C) transfection in mammary carcinoma cells. Relative expression of (A) TLR3, (B) Ifih1/MDA5, (C) Ddx58/RIG-I, (D) Zbp1, (E) Ddx60, (F) Dhx9, and (G) Ifi204. (***p < 0.001, **p < 0.01, * p < 0.05, ns = non-significant, n = 3–5).

**Figure 6.**
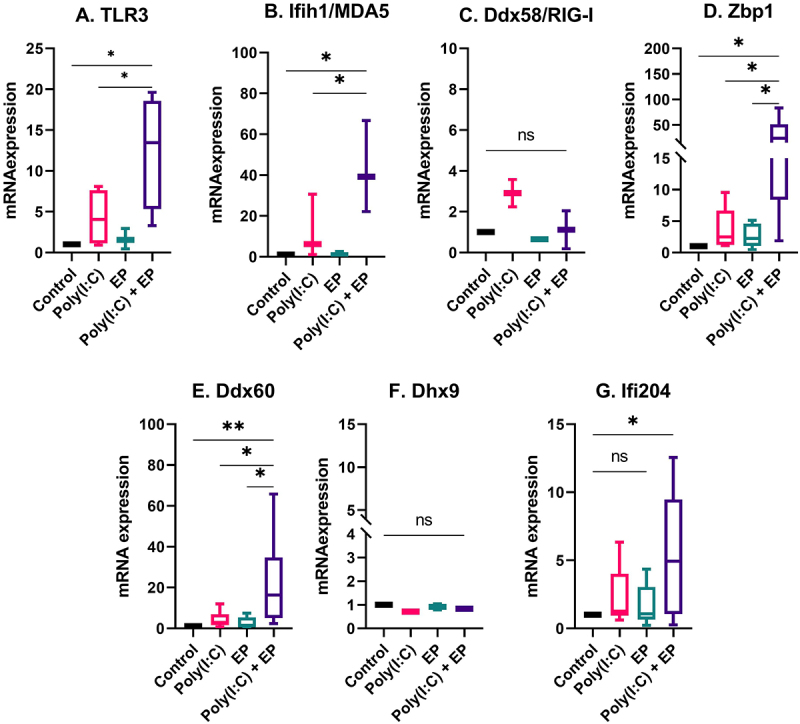
The regulation of several dsRNA sensor and co-sensor mRNAs by poly(I:C) transfection in fibrosarcoma cells. Relative expression of (A) TLR3, (B) Ifih1/MDA5, (C) Ddx58/RIG-I, (D) Zbp1, (E) Ddx60, (F) Dhx9, and (G) Ifi204. (***p < 0.001, **p < 0.01, * p < 0.05, ns = non-significant, n = 3–5).

The fact that Type I IFN and pro-inflammatory cytokine and chemokine mRNAs were upregulated and pro-inflammatory proteins were secreted implicated the activation of RNA sensing by poly(I:C) transfection [6,10]. We therefore evaluated the mRNA levels of the dsRNA sensors TLR3, Ifih1/MDA5, Ddx58/RIG-I, and Zbp1 and the co-sensors Ddx60, Dhx9, and Ifi204 by RT-qPCR. The mRNAs of Ddx58/RIG-I and Dhx9 were detected in each cell line but were not regulated (Figs. 5 and 6). However, the mRNAs of TLR3, Ifih1/MDA5, Zbp1, Ddx60 and Ifi204 were upregulated by varying degrees after poly(I:C) transfection in both mammary carcinoma (Fig. 5) and fibrosarcoma (Fig. 6) cells. The level of upregulation was similar between the two cells types.

### The regulation of components of the RNA sensing signal transduction pathways

**Figure 7.**
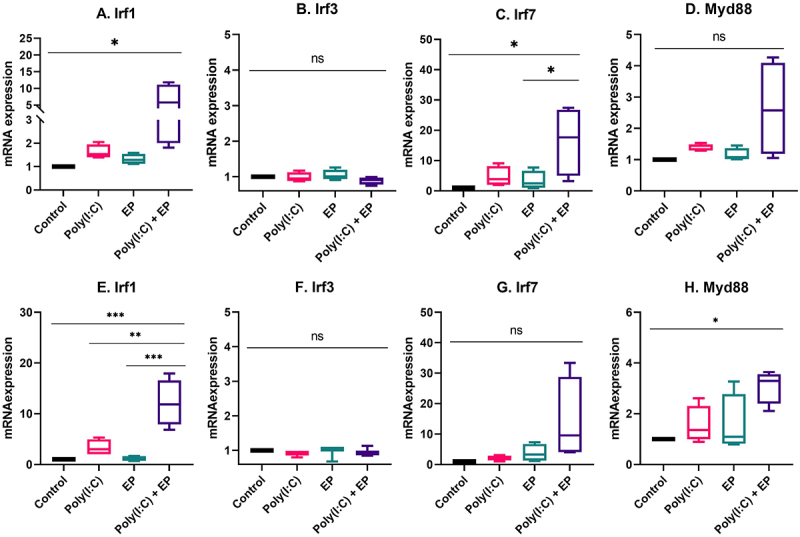
The regulation of interferon regulatory factor mRNAs after poly(I:C) transfection. Relative mRNA expression of (A) IRF1, (B) IRF3, (C) IRF7 and (D) MyD88 in mammary carcinoma cells; (E) IRF1, (F) IRF3, (G) IRF7 and (H) MyD88 in fibrosarcoma cells. (***p < 0.001, * p < 0.05, ns = non-significant, n = 3–5).

We next evaluated the mRNA expression levels of several molecules important to RNA sensing and the pro-inflammatory response (Fig. 7). IRF1 contributes to IFNβ transcription [25,26] and expression is increased by treatment with poly(I:C) [27]. As expected, this mRNA was upregulated after poly(I:C) transfection in mammary carcinoma (p < 0.05) and fibrosarcoma cells (p < 0.001) when compared to control. Several RNA sensors, including TLR3, Ifih1/MDA5, Ddx58/RIG-I, Zbp1, Ddx60 and Dhx9 signal via IRF3 and/or IRF7 [6,10]. These mRNAs were detected in each cell line, but differentially upregulated. IRF3 was not regulated, while IRF7 was upregulated in mammary carcinoma cells (p < 0.05). In DNA sensing, Dhx9 signals via MyD88 [7]. This mRNA was not upregulated in mammary carcinoma cells, but was upregulated in fibrosarcoma cells (p < 0.05) by poly(I:C) transfection.

**Figure 8.**
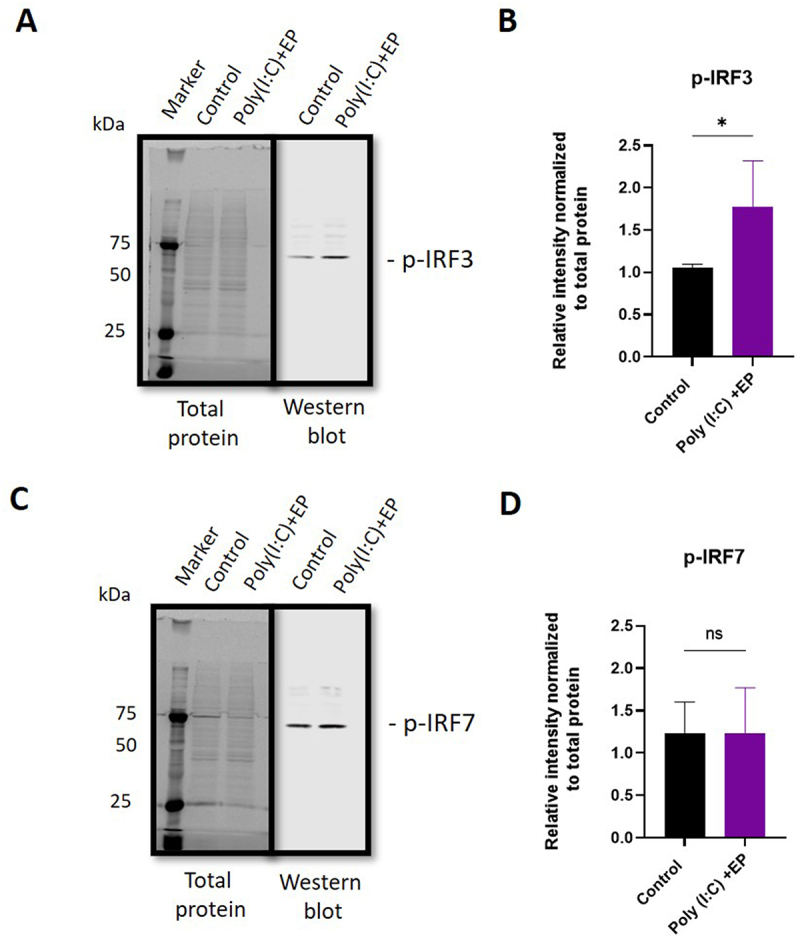
Quantification of phosphorylated interferon regulatory factor protein in mammary carcinoma cells using western blots. (A) representative image of phosphorylated IRF3 blot shown with total protein stain, (B) phosphorylated IRF3 protein quantification, (C) representative image of phosphorylated IRF7 blot shown with total protein stain, (D) phosphorylated IRF7 protein quantification. (n = 4). *, p < 0.05.

Messenger RNA upregulation does not necessarily translate to increased protein activity. We quantified phosphorylated and therefore transcriptionally active IRF3 and IRF7 in mammary carcinoma cells using western blots (Fig. 8 and Supplementary Figure 1). With poly(I:C) electroporation, the levels of phosphorylated IRF3 increased (p < 0.05), while no increase in IRF7 phosphorylation was detected.

## Discussion

In this study, we tested a carcinoma and a sarcoma cell line to determine whether these varying tumour cell types would respond to poly(I:C) delivery via electroporation. We found that the effect of poly(I:C) on cell death was amplified by transfection. The levels of mRNAs or proteins of several pro-inflammatory molecules were also amplified after poly(I:C) electroporation. The mRNAs of several RNA sensors were present and several of them, and several of them were significantly upregulated. Components of RNA sensing signal transduction pathways were present or regulated. These observations and the fact that intratumour DNA delivery has been developed preclinically as a cancer therapy and is now in clinical trials for solid tumours [20,21] support the concept that delivery of an alternative nucleic acid, poly(I:C), by electroporation could become an effective sole or combination solid tumour therapy.

Cell death is a potential outcome of RNA sensing [6,10]. We did not observe cell death in either cell type with simple short-term poly(I:C) exposure (Fig. 1). Our findings confirmed previous studies in breast cancer cell lines where simple exposure was insufficient to induce cell death [28,29]. We did detect significant cell death after poly(I:C) electroporation. Cell death, generally by apoptosis, in breast cancer cell lines has been described after culture with poly (I:C) [30] or with a transfection reagent or nanoparticle delivery [31–36]. In a previous comparison of pulse protocols, we found that the viability of fibrosarcoma cell viability was less affected by these pulse conditions than the other pulsing conditions tested [37]. However, we still observed a significant pulse effect in each cell type. Poly(I:C) transfection by electroporation confirmed the increased cell death observed in the studies using other non-viral transfection methods.

Poly(I:C) mimics viral infection and elicits the secretion of type I IFN and pro-inflammatory cytokines, which play an essential role in inducing immune response activation (Yu and Levine 2011). We found that the mRNAs of the pro-inflammatory molecules Cxcl10 and IL-6 were upregulated in both cell types, while TNFα mRNA was upregulated only in mammary carcinoma cells when cells were transfected with poly(I:C) (Fig. 2). We also detected significant secretion of IFNβ and IL-6 by both cell types by ELISA (Fig. 3) and the secretion of chemokines CCL2, CCL3, CCL4 and CXCL10 by both cell types (Fig. 4) in the same experimental group. Interestingly, short-term poly(I:C) exposure was adequate to induce significant CCL2 secretion by fibrosarcoma cells. Type I interferon production is a marker for PRR activation and several studies have demonstrated type I interferon secretion by breast carcinoma [30,33,38,39] or sarcoma [40] cells after poly(I:C) exposure. The pro-inflammatory cytokine IL-6 is secreted by Kaposi’s sarcoma cells in response to 24 hours’ exposure to poly(I:C) [41,42]. Finally, a variety of chemokines are induced by poly(I:C) exposure, including Ccl2, Ccl5 and Cxcl1 in 4T1 mammary carcinoma cells in a dose-dependent manner [28]. However, poly(I:C) alone did not induce IFNβ, IL-8, Ccl5, or Cxcl10 mRNAs in a panel of six human breast carcinoma cell lines [29]. Kaposi’s sarcoma cells released IL-6, IL-8, Ccl5, and Cxcl10 in response to long-term poly(I:C) exposure [42]. Based on these previous studies, our results specifically confirm mRNA upregulation or secretion of the pro-inflammatory molecules IFNβ, IL-6, Ccl2, and Cxcl10.

Pro-inflammatory signalling implicates the activation of RNA sensing by the dsRNA synthetic analogue poly(I:C). Although many RNA sensors are unassigned, several currently known RNA sensors are classified as TLRs, RLRs, and ALRs [1]. RNA helicases are primary factors in RNA metabolism, and they also play critical roles in innate immune sensing. These sensors can evoke innate immune responses by activating kinases, adaptors, and signalling transduction proteins, triggering the induction of type I IFN and various pro-inflammatory factors. To reveal potential RNA sensors involved in poly(I:C)-induced immune activation in mouse mammary carcinoma and fibrosarcoma cells, we analysed RNA sensor mRNA levels. We found that each RNA sensor or co-sensor mRNAs we tested was expressed in each cell type, including TLR3, Ifih1/MDA5, Ddx58/RIG-I, Zbp1, Ddx60, Dhx9, and Ifi204 (Figs. 5 and 6).

The dsRNA sensor TLR3 is expressed in the endosomes of the cell [2]. Like many non-viral delivery methods, electroporation delivers nucleic acids into cells via an endocytosis-like mechanism [43,44]. These observations make TLR3 a likely candidate for poly(I:C) sensing. Previous studies demonstrate that TLR3 is expressed in breast carcinoma [29] and Kaposi’s sarcoma [42] cells. We found that the mRNA was expressed in both cell lines and upregulated in response to poly(I:C) electroporation.

The RLRs Ifih1/MDA5 and Ddx58/RIG-I function as cytoplasmic sensors of viral RNA [45] and have anticancer roles [46]. Ifih1/MDA5 mRNA was upregulated after poly(I:C) transfection of each cell type; however, Ddx58/RIG-I mRNA, although present, was not upregulated. This may be because Ddx58/RIG-I is selective for shorter dsRNA sequences than Ifih1/MDA5 [47]. Interferon-inducible protein 16, the human ortholog of mouse ALR Ifi204, senses dsRNA and enhances Ddx58/RIG-I transcription [9]. We found that the mRNA for Ifi204 was upregulated after poly(I:C) electroporation. We previously reported that Ifi204 was responsible for controlling type I IFN signalling after DNA electroporation [48]; this protein may also be important to in poly(I:C) signalling. Zbp1 recognizes influenza A virus genomic RNA and to trigger cell death [5]. We previously demonstrated that Zbp1 is highly upregulated after electroporation of plasmid DNA and in fact binds plasmids intracellularly [48]. Here, we observed the upregulation of Zbp1 mRNA in each cell type after poly(I:C) transfection. Our results highlight the role of Zbp1 in the RNA sensing. There are two proposed mechanisms by which DEAD-box or DEAH-box helicases participate in RNA sensing; they act independently of other RNA sensors or serve as co-sensors of RLRs or NLRs to potentiate their activation [10]. Ddx60 interacts with RLRs to facilitate the induction of type I IFNs and interferon-stimulated genes [49]. Dhx9, a member of the DExD/H-box family of helicases with a ‘DEIH’ sequence at its eponymous DExH-box motif, was expressed in both cell lines but not upregulated in the presence of poly(I:C) or when poly(I:C) was electroporated into the cells. Dhx9 interacts with Interferon beta promoter stimulator protein 1 (Mitochondrial antiviral-signalling protein or MAVS) in myeloid dendritic cells and controls production of type I IFN and pro-inflammatory cytokines [50]. Similar to Zbp1, we also demonstrated that, although the mRNA is not upregulated, Dhx9 physically binds plasmid DNA after electroporation [48]. It is interesting to note that Ifih1/MDA5, Ifi204, Ddx60, and Zbp1 are known to be interferon-inducible and this upregulation may be important functionally. Ddx58/RIG-I is also interferon inducible but, in spite of the detection of IFNβ secretion, the mRNA was not regulated in our study.

Finally, RNA sensing signal transduction pathways extensively overlap with potential cross-talk between sensors, making it difficult to predict specific outcomes. We chose to assay several representative signalling pathway proteins. IRF1 protein induces transcription of interferon stimulated genes [25,26]. Several RNA sensors signal via IRF3 or IRF7 homodimers or IRF3/IRF7 heterodimers. Dhx9 senses cytosolic nucleic acids using MyD88 as a downstream adaptor [51]. We therefore quantified the mRNAs of each of these signalling proteins. In breast cancer cells, IRF3 and MyD88 mRNAs were detected while IRF1 and IRF7 mRNAs were upregulated (Fig. 7). The observation that IRF3 was constitutively expressed while IRF7 is inducible in Type I interferon gene expression supports previous studies [52]. The regulation of IRF7 mRNA confirms a previous study in with poly(I:C) in breast cancer cells [53]. Increased levels of phosphorylated IRF3 but not IRF7 were detected after poly(I:C) electroporation in mammalian carcinomas (Fig. 8). In fibrosarcoma cells, IRF3 and IRF7 mRNAs were detected, while IRF1 and MyD88 mRNAs were regulated (Fig. 7). While TLR3 signals vis IRF3, Ifih1/MDA5 and Ddx58/RIG-I signal via both IRF3 and IRF7 [54]. In DNA sensing, ZPB1 signals via both IRF3 and IRF7 [55] and therefore this pathway may be activated in RNA sensing. Based on current knowledge on nucleic acid sensing, it is likely that the activation of endosomal TLR3, cytosolic Ifih1/MDA5 and/or potentially cytosolic ZBP1 is possibly responsible for IRF3 activation.

PRR activation induces the innate immune system, which may potentiate adaptive immunity. The coordinated release of cytokines could intensify an inflammatory response to increase tumouricidal activity. Consequently, ligands for PRRs such as poly(I:C) and CpG motif DNA have been used as analogues of pathogen RNA and DNA, respectively, to create immunomodulators as sole or combination cancer therapies.

The function of poly(I:C) in tumour immunotherapy has been explored for several decades. In the 1970s, studies in mammary carcinoma preclinical models demonstrated some success [56,57], but these results were not always confirmed [58]. By the 1980s, clinical trials were initiated [59]. A recently published clinical trial combined a peptide vaccine with a poly(I:C) derivative for early-stage breast cancers [60]. As clinical trials were initiated for breast cancer, testing was also underway in sarcoma models [61]. Poly(I:C) derivatives have also reached clinical trials for sarcoma [62]. In current clinical trials, poly(I:C) derivatives are being tested as sole or combination therapies for many solid tumour types (www.clinicaltrials.gov).

## Conclusions

We report that poly(I:C) transfection decreased cell viability in both mammary carcinoma and fibrosarcoma cell lines with a concomitant pro-inflammatory response. Electroporated Poly (I:C) stimulated the mRNAs of several sensors and signalling pathway proteins. In the future, determining the contribution of the emerging RNA sensors to their direct RNA-sensing roles in the cell metabolism as well as the mechanism by which different RNA sensors cross-talk will enhance our understanding of how RNA sensing and metabolism and their activities cooperate in the induction of nucleic acid-induced immunity.

## Supplementary Material

Supplemental Material
