# Supplementary material for: Poly(I:C) transfection induces a pro-inflammatory cascade in murine mammary carcinoma and fibrosarcoma cells
Source: RNA Biol. 2022 Jun 23;19(1):841–51. doi: 10.1080/15476286.2022.2084861 (PMC9235898; doi:10.1080/15476286.2022.2084861)
Supplement: Supplemental Material [file KRNB_A_2084861_SM5377.zip › Supplementary Table 1.pdf]

Supplementary Table 1. RT-qPCR primer sequences

**Target**	Forward Primer	Reverse Primer
**Reference Genes**		
β Actin	GAAGTGTGACGTTGACATCC	ACTCATCGTACTCCTGCTTG
GAPDH	TTCACCACCATGGAGAAGGC	GGCATGGACTGTGGTCATGA
**Pro-inflammatory Markers**		
CXCL10	TCAGCACCATGAACCCAAG	CTATGGCCCTCATTCTCACTG
IL6	AAACCGCTATGAAGTTCCTCTC	GTGGTATCCTCTGTGAAGTCTC
TNFα	CCCTCCAGAAAAGACACCATG	GTCTGGGCCATAGAACTGATG
RNA sensors and co-sensors		
TLR3	GAGAACCTCCAAGAACTGCTC	ATTGTCTGGAAACACCCCG
Ifih1/MDA5	CCTTGAAAACTCCCTCTTAAATCT	GCTTCTGCTTCAAATATCGTCTC
Ddx58/RIG-I	GCTGCCATGCAGAGTGATTGGAAA	ACTCTTGGCCACACAGCTGTAGAA
Zbp1	TGCTTTCTAGAGGACGCCACCATT	TGGCTTCAGAGCTTGTACCTGTGT
Ddx60	ACTGGAACACTCGCTTTGG	GAAGTAGACATCACCCAACAGG
Dhx9	AAACTCCCCATTGAACCTCG	TGTATCCCAGGCGTTTTCC
Ifi204	CCAGTCACCAATACTCCACAG	GAGCACCATCACTGTCAGG
Signal Transduction		
IRF1	GGAAGCTGTGTGGAGATGTTAG	TAGCCCTGAGTGGTGTAACT
IRF3	GTCTTAAGGAGCTGTTAGAGATGG	TGGTCAGAGGTAAGGGAGATAG
IRF7	CTTCTCCAGCATGTGTCTCC	CCTACCTCCCAGTACACCTT
Myd88	AACAAAGGAACTGGGAGGC	GTCTGTTCTAGTTGCCGGATC
