# Supplementary figures and images for: Poly(I:C) transfection induces a pro-inflammatory cascade in murine mammary carcinoma and fibrosarcoma cells
Source: RNA Biol. 2022 Jun 23;19(1):841–51. doi: 10.1080/15476286.2022.2084861 (PMC9235898; doi:10.1080/15476286.2022.2084861)

Supplementary Figure 1. Original western blots.

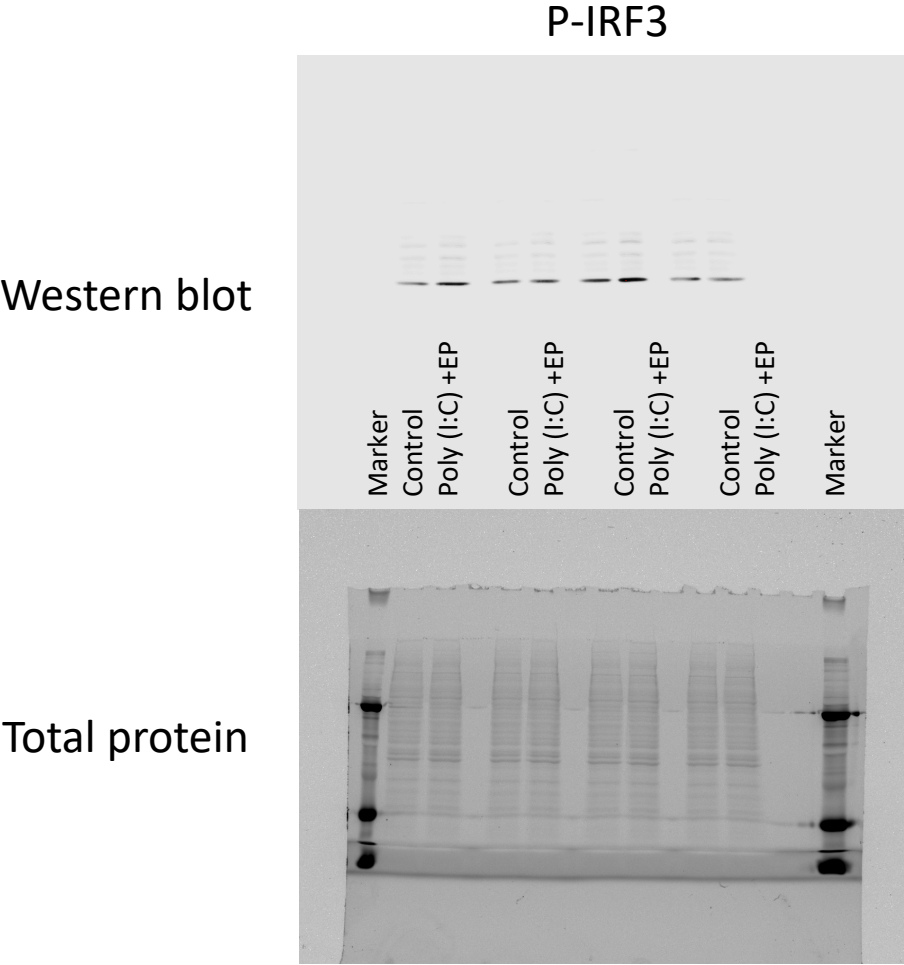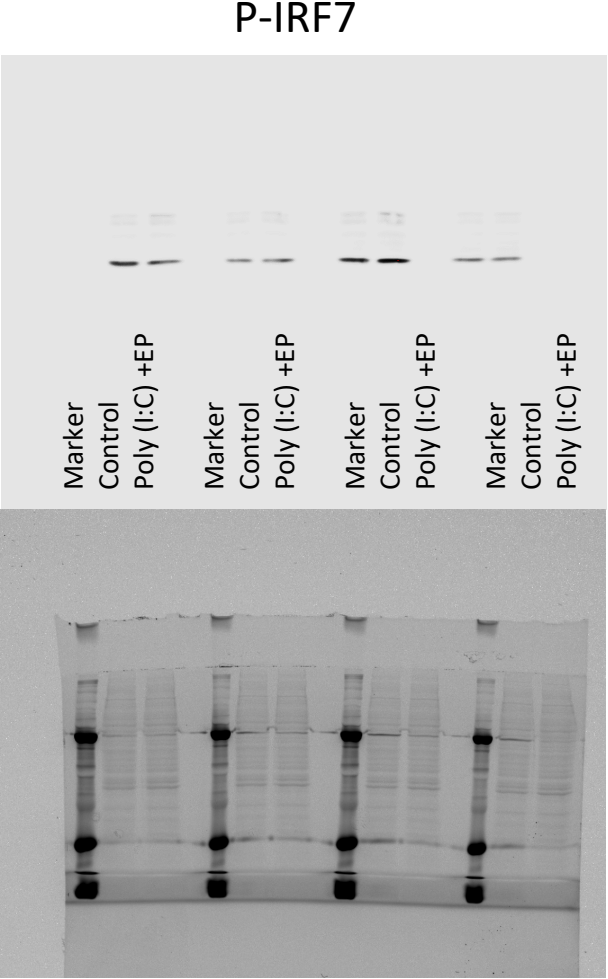

Supplement: Supplemental Material [file KRNB_A_2084861_SM5377.zip › Supplementary Figure 1.pdf]
